# A Case Report of Localized Vegetative Pyoderma Gangrenosum of the Foot in a Patient With Myasthenia Gravis in an Outpatient Setting

**DOI:** 10.7759/cureus.41181

**Published:** 2023-06-30

**Authors:** Zeib Syed, Shahrukh Syed, Katherine Burmaster

**Affiliations:** 1 Internal Medicine, LewisGale Medical Center, Roanoke, USA; 2 Internal Medicine, Edward Via College of Osteopathic Medicine (VCOM), Blacksburg, USA; 3 Internal Medicine, LewisGale Medical Center, Salem, USA

**Keywords:** dermatology, vegetative pyoderma gangrenosum, myasthenia gravis, pathergy, pyoderma gangenosum

## Abstract

Pyoderma gangrenosum (PG) is a reactive, non-infectious inflammatory neutrophilic dermatoses that have historically presented as a diagnostic and therapeutic dilemma. It is often misdiagnosed as other disease processes, particularly ulcers, and is thus associated with a delay in care. Pyoderma gangrenosum, left untreated, carries a three times mortality risk compared to the general population. There are multiple subtypes and presentations reflected in the current research which shows that there is still much to be understood about this disorder. Here we examine the unique presentation of a vegetative type of pyoderma gangrenosum through the case of a 69-year-old male that presents with a persistent lesion on his foot.

## Introduction

Pyoderma gangrenosum is a rare inflammatory skin disease characterized by neutrophilic infiltration of the skin and is often mistaken for other vascular, malignant, infectious, or inflammatory disorders. Accurate identification and exclusion of other pathologies and adequate treatment are imperative to accurately treat patients and decrease morbidity in patients. At present estimates, this disease impacts about three to 10 million people annually. Pyoderma Gangrenosum is generally thought to present as a persistent, sterile, and deep ulceration involving the lower extremities and can even be associated with systemic diseases such as rheumatoid arthritis, hematological malignancies, and hepatic pathologies. Ulcerative colitis and Crohn's disease may also be found [1, 2]. There are four postulated subtypes; pustular pyoderma gangrenosum, bullous pyoderma gangrenosum, vegetative pyoderma gangrenosum, and ulcerative pyoderma gangrenosum. The vegetative subtype of this disease is usually heralded as the least typical and usually presents as a superficial ulcer that does not support the typical purple edge and is typically not associated with other underlying systemic conditions [3]. Left untreated, this disease typically carries three times the mortality risk compared with the general population [2]. Rapid recognition and treatment are imperative in the management of cases of pyogenic gangrenosum [4]. Here we examine the case of a 69-year-old male with a history of myasthenia gravis that presents with a lesion morphologically consistent with vegetative pyoderma gangrenosum.

## Case presentation

The patient is a 69-year-old male that has been lost to medical follow-ups for several years presenting to the outpatient clinic for a regular physical and to establish care. He presented with complaints of pain and discomfort with ulceration on the right foot. He reports living an active lifestyle and generating a high volume of movement in his daily life and subsequently reports his current footwear exacerbates his right foot lesion. To emphasize the point, he reports that he walks about 20-30 miles per day to commute to work. As he has been out of the medical system for some time, he had been taking a tweezer and needle, which he reports to have sterilized, to periodically drain this lesion of serosanguinous fluid. Despite his efforts, the lesion persisted for several years.

**Figure 1:**
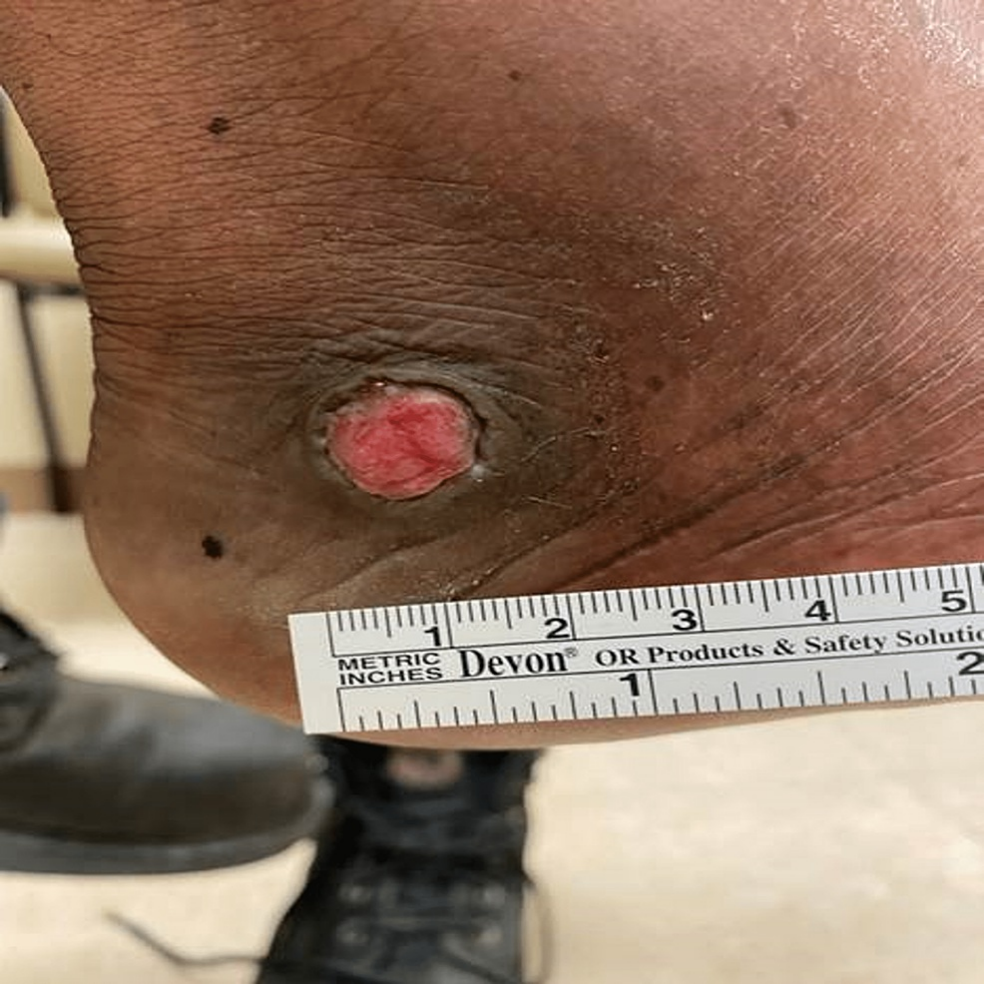
Vegetative pyoderma gangrenosum of the right lower extremity measuring at 1.5 cm and presenting as a tender pink cap-like lesion with pale rolled borders.

Physical examination revealed a 1.5 cm pink cap-like lesion with pale rolled purple borders on the right foot located just inferior to the lateral malleolus (Figure 1). He reported mild tenderness upon palpation and the lesion was notably without significant redness or purulent expression. He reported a slow progression of the lesion over time. The patient had callouses at both great hallux and flattened arches of the right foot.

The patient was referred to dermatology for a biopsy of the lesion and a topical clobetasol 0.05% was prescribed to apply twice a day for his solitary lesion. A screening colonoscopy order was set to investigate his ongoing pyoderma gangrenosum. Patient unfortunately was lost to follow-up despite multiple outreach attempts. The goal was for patients lesion to regress by the time we had seen him again otherwise an escalation in therapy would be warranted.

## Discussion

Pyoderma gangrenosum is most commonly associated with inflammatory bowel disease and this relationship has been well established in literature. It has also been demonstrated that the disease course of pyoderma gangrenosum does not necessarily have to reflect the disease course of inflammatory bowel disease [5]. With progressing research, the pathophysiology and subsequent treatment look at immune dysregulation involving both adaptive and innate immunity [6]. Although rare, there have been prior presentations similar to this case in which patients had underlying myasthenia gravis which had been shown to be affiliated with pyoderma gangrenosum although noted to be rare [7]. There have been postulated paraneoplastic autoimmune-mediated diseases associated with malignancies which have previously been described in reviews to include both myasthenia gravis and pyoderma gangrenosum in the setting of cancer [8].

Morphologically, we observe what appears to be the vegetative form of pyoderma gangrenosum which is a particularly rare superficial variant of the disease. The vegetative subtype is one of the least common manifestations of the disease. It usually presents as a superficial ulcer with non-purulent bases and no surrounding erythema and typically impacts the head and neck [9, 10]. It is most typically found on the trunk as single or multiple lesions and usually is not affiliated with systemic disease [11]. In the case of our patient, he had only one solitary lesion on his right foot.

In our case, the patient has had a poor follow-up with the healthcare system and a known history of myasthenia gravis presenting with a lesion that appears to be vegetative pyoderma gangrenosum. His lesion has a unique presentation in that he has an underlying myasthenia gravis and that his solitary lesion is located on his lower extremities. The patient estimates that he walks about 20 miles a day and there seems to be a component of pathergy with the patient’s footwear and inordinate ambulation [1].

This case is atypical as this patient has a very rare vegetative form of pyoderma gangrenosum with localization to the lower extremities and possible pathergy in the setting of underlying myasthenia gravis. The patient started treatment with a topical steroid and requested to return for a follow-up. Unfortunately, this patient had been lost to follow-up despite multiple attempts at following up. Given the association of pyoderma with paraneoplastic autoimmunity and underlying cancers as well as underlying inflammatory bowel disease we urged the patient to follow up with GI as well to further delineate the underlying cause of this patient’s disease.

## Conclusions

Pyoderma Gangrenosum is a rare condition that is not seen often and much remains to be understood regarding disease pathophysiology and associations. Untreated, the condition can lead to more extensive tissue damage and has been noted to be associated with increased mortality when compared to the general population. Here we see an atypical presentation with unique distribution of vegetative pyoderma gangrenosum with associated pathergy in the setting of myasthenia gravis in an older gentleman with poor healthcare follow-up. We also demonstrate the incidence of the vegetative subtype for the first time in the setting of myasthenia gravis. In order to more efficiently diagnose these cases and treat them adequately, a greater understanding of their presentations and associations should be known.
